# New insights into the interaction between m6A modification and lncRNA in cancer drug resistance

**DOI:** 10.1111/cpr.13578

**Published:** 2023-11-14

**Authors:** Yizhou Jin, Zhipeng Fan

**Affiliations:** ^1^ Beijing Key Laboratory of Tooth Regeneration and Function Reconstruction, Beijing Stomatological Hospital, School of Stomatology Capital Medical University Beijing China; ^2^ Beijing Laboratory of Oral Health Capital Medical University Beijing China; ^3^ Research Unit of Tooth Development and Regeneration Chinese Academy of Medical Sciences Beijing China

## Abstract

Drug resistance is perhaps the greatest obstacle in improving outcomes for cancer patients, leading to recurrence, progression and metastasis of various cancers. Exploring the underlying mechanism worth further study. N6‐methyladenosine (m6A) is the most common RNA modification found in eukaryotes, playing a vital role in RNA translation, transportation, stability, degradation, splicing and processing. Long noncoding RNA (lncRNA) refers to a group of transcripts that are longer than 200 nucleotides (nt) and typically lack the ability to code for proteins. LncRNA has been identified to play a significant role in regulating multiple aspects of tumour development and progression, including proliferation, metastasis, metabolism, and resistance to treatment. In recent years, a growing body of evidence has emerged, highlighting the crucial role of the interplay between m6A modification and lncRNA in determining the sensitivity of cancer cells to chemotherapeutic agents. In this review, we focus on the recent advancements in the interaction between m6A modification and lncRNA in the modulation of cancer drug resistance. Additionally, we aim to explore the underlying mechanisms involved in this process. The objective of this review is to provide valuable insights and suggest potential future directions for the reversal of chemoresistance in cancer.

## Graphical abstract

In the past, most studies concerning m6A modification focused on mRNAs. Transcriptome‐wide mapping technology reveals a significant presence of m6A‐modified lncRNA, which has been shown to play vital roles in biological processes. Interestingly, the expressions and functions of m6A modification are also regulated by lncRNA in turn. The roles of m6A in the regulation of lncRNA mainly concentrate in following aspects: (1) Change the structure of lncRNA and affect its interaction with proteins, known as ‘m6A switch’. (2) Mediate gene transcription repression. (3) Mediate a competing endogenous RNA (ceRNA) model. (4) Regulates lncRNA stability or degradation. However, lncRNA has an impact on m6A regulators as well. LncRNA can influence the stability and degradation of m6A‐related enzyme proteins or combine with them to form interaction complexes, thus facilitating a regulatory effect on the downstream target mRNA of m6A regulators. Drug resistance is perhaps the greatest obstacle in improving outcomes for cancer patients, leading to recurrence, progression and metastasis of various cancers. In this review, we summarize all the identified interactions between m6A modification and lncRNA that participate in chemoresistance, aiming to provide a novel insight into the cancer treatment.

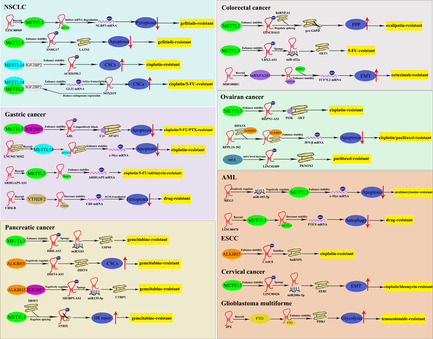

## 1 INTRODUCTION

**FIGURE 1:**
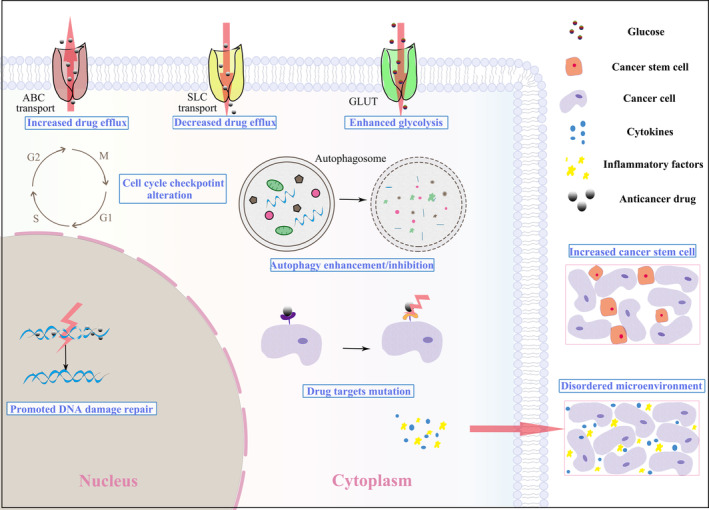
Mechanisms underlying chemoresistance in cancer. Increased/decreased drug efflux leads to decreased therapeutics accumulation in cancer cells; Promoted DNA damage repair, increased cancer stem cells and enhanced autophagy protect cancer cells from anticancer drugs; Cancer cells prefer consuming glucose to produce lactic acid, known as ‘aerobic glycolysis’; Disordered tumour microenvironment, alteration of cell cycle checkpoint and drug targets will also reduce the efficacy of therapeutic drugs.

More than 600,000 people die from cancer each year, which is a threatening challenge that needs to be solved.^1^, ^2^ Traditional oncotherapy typically encompasses surgical resection, chemotherapy, and radiotherapy. Several novel treatment strategies have emerged in recent years, including biological immunotherapy, gene therapy, and targeted therapy.^3^, ^4^ Chemotherapy is one of the most effective treatment strategies for cancer, making it the first choice for patients with advanced and recurrent cancer. The resistance to chemotherapeutics poses a serious obstacle for cancer treatment, leading to a decrease in the efficacy of these drugs and resulting in poor prognosis for cancer patients. It is necessary to clarify the underlying mechanisms and identify novel treatment targets or therapeutic sensitizers. Generally, chemoresistance can be classified into two main categories: intrinsic resistance and acquired resistance.^5^, ^6^ Intrinsic resistance refers to resistance induced by factors that exist prior to the administration of any treatment, while acquired resistance is the result of adaptive responses that protect tumour cells from unfavourable environments during drug treatment.^7^, ^8^, ^9^, ^10^ Multiple mechanisms have been demonstrated to underlie chemoresistance, encompassing impaired drug transport systems, altered metabolism patterns, and mutations in drug targets.^11^, ^12^, ^13^, ^14^ Additionally, the augmentation of cancer stem cells (CSCs), enhancement of DNA damage repair ability, induction of tumour cell autophagy and alteration of the tumour immune microenvironment (TME) are also significant factors responsible for the reduction in the efficacy of antitumor drugs^12^ (Figure 1).

N6‐methyladenosine (m6A) modification is one of the most abundant modification found in eukaryotic messenger RNA (mRNA) and has been demonstrated involving in various biological processes, including RNA splicing, translation, and degradation.^15^, ^16^, ^17^, ^18^ The process of m6A modification is dynamic and reversible, which is regulated by methylases (‘writers’) and demethylases (‘erasers’). m6A writers encompass a group of proteins that includes METTL3,^19^ METTL14,^20^ WTAP,^21^ RBM15,^22^ KIAA1429,^23^ METTL16,^24^ and ZC3H13.^25^ While m6A modification is removed by erasers including FTO and ALKBH5.^26^, ^27^ m6A reader proteins are responsible for recognizing RNA m6A modification sites. The primary m6A reader protein contains YTH domain,^28^ including YTHDC1 located in the nucleus and YTHDC2, YTHDF1, YTHDF2, and YTHDF3 located in the cytoplasm. Other m6A reader proteins, such as HNRNPs,^29^ IGF2^30^ and eIF3,^31^ have the ability to influence RNA destiny and cellular function by selectively recognizing m6A sites. Some recent advances have revealed a close relationship between m6A modification and various human diseases, with a particular emphasis on cancer drug resistance.^16^, ^20^, ^21^

The m6A modification is not limited to mRNA, but is also found in non‐coding RNAs, such as lncRNA. Long non‐coding RNA (lncRNA) is generally defined as transcripts longer than 200 nucleotides (nt) and lacking the ability to encode proteins.^32^, ^33^ Nevertheless, recent advancements have revealed that certain lncRNA have the ability to encode microproteins, which plays a crucial role in various physiological and pathological activities.^33^ LncRNA exerts significant roles in various biological processes, including transcriptional regulation, nuclear domains organization and regulation of proteins and RNAs.^34^, ^35^ Extensive investigation has revealed a multitude of differentially expressed lncRNA in the transcriptome of chemoresistant cancer cells, as evidenced by numerous studies.^36^, ^37^, ^38^, ^39^ With the advent of MeRIP‐m6A‐seq technology,^40^ the identification of m6A‐modified lncRNA has gained significant attention in clinical research and medical development, especially in the field of oncology.

Therapeutic strategy, which is based on the understanding of molecular mechanisms underlying chemoresistance, results in effective treatments outcomes against molecular targets. EGFR in lung cancer, BCR‐ABL in chronic myeloid leukaemia, BRAF in melanoma, HER2 in breast cancer, and FGFR in various types of cancer are the most well‐studied molecular targets. Some well‐known anticancer drugs against these targets have been designed and applied in clinic treatment.^41^ Exploring the role of lncRNA or m6A‐modified lncRNA in chemoresistance holds the potential to identify novel molecular targets for clinical cancer treatment. In this review, we aim to offer a fresh perspective on cancer treatment by thoroughly summarizing the advancements in research regarding the interaction between m6A modification and lncRNA in the context of cancer drug resistance.

## 2 MUTUAL REGULATION BETWEEN M6A MODIFICATION AND LNCRNA

**FIGURE 2:**
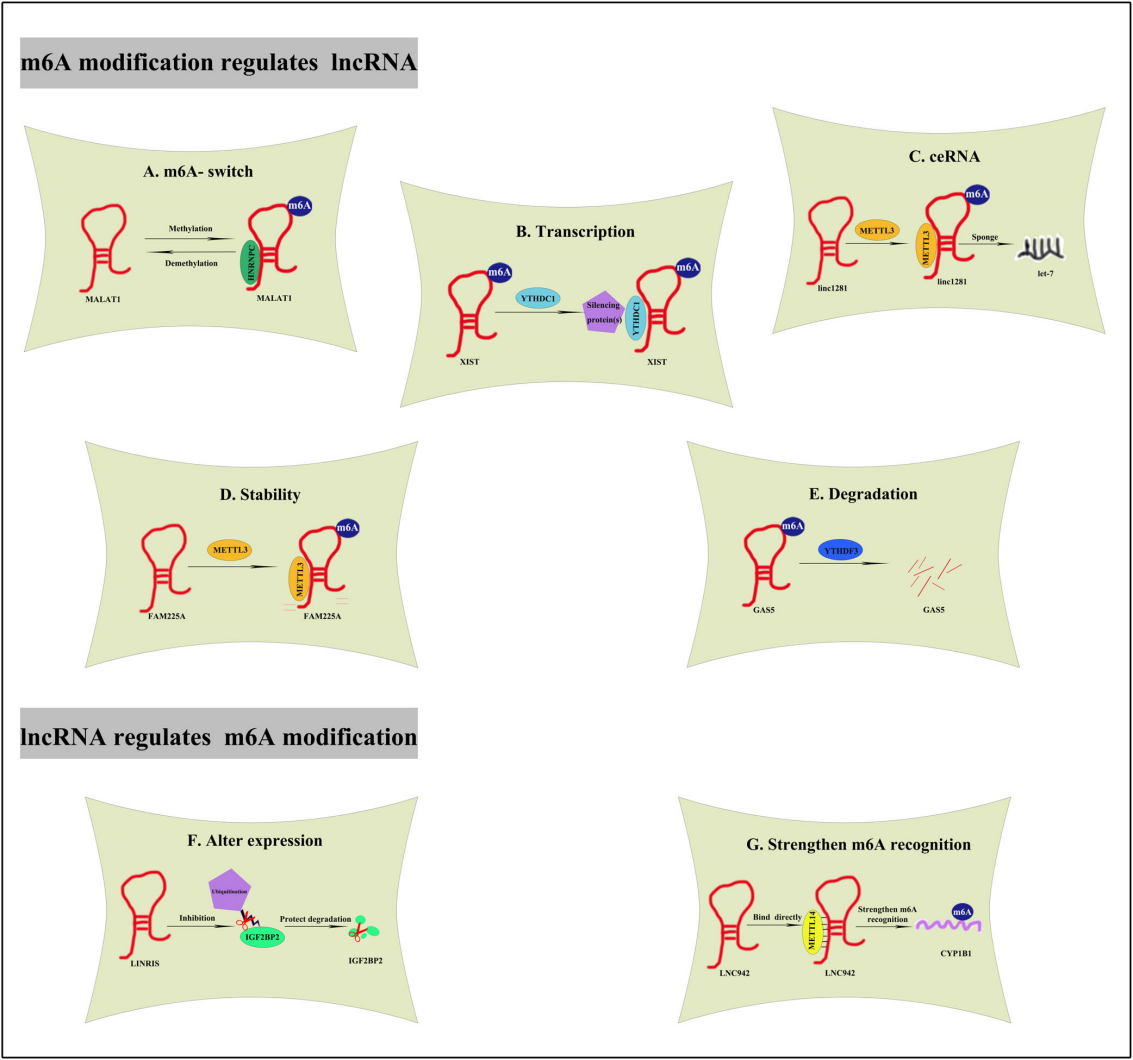
Mutual regulation between m6A modification and lncRNA. (A) m6A modification affects MALAT1 structure and regulates the binding of HNRNPC to MALAT1. (B) YTHDC1 binds to XIST, thus promoting XIST‐mediated gene repression. (C) METTL3‐induced m6A modification helps build Linc1281‐mediated ceRNA model. (D) METTL3‐induced m6A modification improves FAM225A stability. (E) YTHDF3 binds to m6A‐modified GAS5, thus triggering GAS5 degradation. (F) LINRIS blocks K139 ubiquitination of IGFBP2, maintaining its stability. (G) METTL14 directly binds to LNC942, by which strengthen the m6A recognition of its downstream targets including CYP1B1.

In previous research, the majority of investigations pertaining to m6A modification primarily concentrated on mRNAs. These studies aimed to understand the influence of m6A modification on various aspects such as transcript localization, splicing, turnover and translation rates.^42^ Nowadays, the use of transcriptome‐wide mapping techniques has led to the identification of a significant number of m6A‐modified lncRNA. Subsequent studies have demonstrated that these m6A‐modified lncRNA plays vital roles in nucleus, cytoplasm, and extracellular environment.^43^ Interestingly, it has been observed that the expression and function of m6A regulators are reciprocally regulated by lncRNA. LncRNA regulates expression of m6A methyltransferases such as METTL3, METTL14, WTAP, METTL16, as well as demethylases like FTO, ALKBH5, and methyl‐binding proteins including YTHDFs, YTHDCs, IGF2BPs, HNRNPs. These proteins are collectively referred to as ‘m6A regulators’. In this section, we provide a summary of the mutual regulation between m6A modification and lncRNA (Figure 2).

### 2.1 Role of m6A modification in the modulation of lncRNA

RNA‐binding proteins (RBPs) decide many cellular processes by directly binding to specific single‐stranded RNA binding motifs (RBM).^44^, ^45^, ^46^ Most currently identified lncRNA has been found to interact with specific RBPs, resulting in the formation of RNA–protein complexes. RBPs regulate the stability, transport, and transcription process of lncRNA, thereby determining the fate and function of lncRNA. m6A readers function as RBPs for lncRNA, playing a crucial role in the regulation of lncRNA degradation and transcription. These readers recognize and target m6A‐modified sites on lncRNA, thereby exerting their regulatory effects. Currently, the roles of m6A modification in deciding lncRNA function and fates mainly concentrate in following aspects: (1) Change the structure of lncRNA and affect its interaction with proteins, commonly referred to as the ‘m6A switch’.^47^, ^48^ (2) Mediate gene transcription repression.^22^ (3) Mediate a competing endogenous RNA (ceRNA) model.^49^ (4) Regulates lncRNA stability or degradation.^50^, ^51^, ^52^

#### 2.1.1 m6A‐switch

RBMs, such as mRNA and lncRNA, can be modified and changed with their local single‐stranded structures, thereby impeding RNA–protein interactions.^53^, ^54^, ^55^, ^56^ Early in 2015, Liu confirmed that the local structure of lncRNA can be modified by m6A modification on its transcript. This modification enhances the accessibility of its paired RBPs. He termed this process that regulated RNA–protein interaction through m6A‐dependent RNA structural remodelling ‘the m6A‐switch’.^47^ MALAT1 is a highly m6A‐modified lncRNA, carrying four m6A motifs.^48^ The MALAT1 hairpin structure includes a modification site (A2577) for m6A and a binding site for HNRNPC consisting of poly‐U sequences. When A2577 is unmethylated, the poly‐U HNRNPC binding domain is hardly accessible. Once A2577 undergoes m6A modification, the hairpin structure of MALAT1 becomes destabilized, leading to the releases of the poly‐U tract. Consequently, this modification enhances the binding capacity of MALAT1 with HNRNPC.^47^ Moreover, it was observed that HNRNPG does not have a direct binding affinity to the MALAT1 m6A site. However, it has the ability to bind to the m6A‐modified MALAT1 (A2515).^47^ Thus, the m6A modification changes the hairpin structure of MALAT1 and regulates its accessibility to HNRNPC/HNRNPG, suggesting an essential role of m6A modification in the remodelling of the structure and the binding ability with RBPs.^57^

#### 2.1.2 Transcription repression

m6A readers possess the ability of recognizing m6A‐modified lncRNA and recruiting downstream transcriptional ribonucleoprotein complexes. Upon binding to the m6A site of lncRNA, the m6A reader facilitates the formation of transcriptional ribonucleoprotein complexes, which in turn can lead to modifications in downstream gene transcription.^58^ This transcriptional modification is different from traditional epigenetic regulation, as it involves the direct binding of m6A regulators to promoters for its modulation. For instance, lncRNA XIST, highly methylated with over 78 m6A residues, regulates transcriptional silencing of genes located on the X chromosome. The m6A modification of XIST helps recruit YTHDC1, which in turn promotes the binding of YTHDC1 to facilitate XIST‐regulated gene repression, such as Gpc4 and Atrx,^22^ highlighting the role of m6A modification in promoting transcription repression induced by lncRNA.

#### 2.1.3 Competing endogenous RNA

Recent evidence suggests that lncRNA may act as competing endogenous RNA (ceRNA) and thus modulates the expression and biological functions of concerning miRNA. Accumulating evidence has extensively documented the crucial role of the lncRNA‐mediated ceRNA pathway in manipulating gene activities. The m6A modification of linc1281 serves as ceRNA and modulates the differentiation of mouse embryonic stem cells (mESCs). Mechanistically, linc1281 ensures mESCs identification by sequestering miRNA let‐7 in a m6A‐dependent manner. Upon knockdown of METTL3 in mESCs, linc1281 exhibits decreased m6A level and fails to bind with let‐7. This suggests that METTL3 is responsible for the m6A methylation of linc1281, as well as the m6A‐mediated ceRNA model.^49^

#### 2.1.4 Stability/degradation

m6A regulators play a vital role in keeping lncRNA stability. In the cytoplasm, YTHDF1, YTHDF2, and YTHDF3 act integrally and cooperatively to modulate the biological processes concerning m6A RNA methylation.^52^ YTHDF2 mediates m6A‐dependent degradation under normal and stress conditions.^59^ YTHDF3 can recognize and directly bind to lncRNA GAS5, thereby facilitating the degradation of GAS5 in a methylation‐dependent manner.^51^ In addition, it has been observed that lncRNA FAM225A exhibits a significant correlation with poor survival in the context of nasopharyngeal carcinogenesis. Mechanical investigation reveals a decrease in the stability of FAM225A following the suppression of METTL3, indicating the involvement of m6A modification in regulating transcripts stability.^50^

### 2.2 Participate of lncRNA in m6A structure and function

More than 28,000 lncRNA transcripts have been identified in human genome so far. LncRNA has emerged as an important regulator of gene expression and multiple cellular processes.^60^, ^61^, ^62^ Recent research has demonstrated that lncRNA may participate in the installation, removal and recognition of m6A modification on RNA molecules. LncRNA can influence the stability and degradation of m6A‐related enzyme protein or combine with them to form interaction complexes, thus facilitating a regulatory effect on the target molecules.^63^

#### 2.2.1 LncRNA alters m6A regulators expression

As an epigenetic regulator involved in various biological processes, lncRNA also plays a role in regulating the mRNA stability of m6A regulators. IGF2BP2 recognizes m6A‐modified mRNAs and maintains the stability of these transcripts through the recruitment of RNA stabilizers.^64^, ^65^ Wang illustrated that the knockdown of lncRNA LINRIS led to the repression of IGF2BP2. Mechanistically, LINRIS inhibits the K139 ubiquitination of IGF2BP2, thus preventing the degradation of IGF2BP2 through autophagy‐lysosome pathway.^63^ In addition, the expression of LNC942 was upregulated in breast cancer cells, accompanied by elevated mRNA stability and increased protein expression of METTL14. Mechanistically, the upregulation of METTL14 was observed upon the overexpression of LNC942, whereas the downregulation of METTL14 was observed upon the knockdown of LNC942 in breast cancer cells.^66^ These evidences fully reveal the role of lncRNA in modulating the expression of m6A regulators.

#### 2.2.2 LncRNA strengthens m6A recognition

LncRNA strengthens the recognition ability of m6A readers by directly binding to specific binding sites. The earliest evidence was documented in year 2020. LINC00266‐1, which encodes a 71‐amino acid peptide, mainly interacts with RBPs, including IGF2BP1. This 71‐amino acid peptide is named ‘RNA‐binding regulatory peptide’ (RBRP). RBRP exhibits direct binding affinity towards IGF2BP1, thereby enhancing the recognition of m6A modifications on RNA molecules. c‐Myc mRNA is one of RBRP's targets. RBRP, together with IGF2BP1, has the potential to enhance the stability and expression level of c‐Myc mRNA, thereby promoting c‐Myc‐mediated tumorigenesis.^30^ In addition, it has been observed that lncRNA MIR100HG could enhance the recognition of m6A modifications on RNA molecules by interacting with hnRNPA2B1, and thus promote the stabilization of TCF7L2 mRNA.^67^ Moreover, LNC42 not only promotes METTL14 expression by enhancing its mRNA stability, but also directly recruits METTL14 protein by binding to a specific recognize sequence. LNC42 recruits METTL14 directly in order to enhance the stability of downstream targets, such as CXCR4 and CYP1B1.^66^ The mentioned evidences thoroughly indicate that lncRNA plays a significant role in enhancing m6A recognition.

To conclude, m6A modification not only regulates the biogenesis and function of lncRNA, but is also affected by lncRNA. There exists a significant correlation and reciprocal regulation between m6A modification and lncRNA.

## 3 MECHANISMS UNDERLYING CANCER DRUG RESISTANCE

Accumulating studies have indicated the vial roles of lncRNA in the proliferation, migration, and invasion of various tumour cells. In recent years, there has been a growing emphasis on the issue of treatment resistance. Evidence suggests that lncRNA affects the sensitivity of tumour treatment through multiple mechanisms. These mechanisms include but are not limited to drug transport modulation, enhancement of DNA damage repair capacity, induction of autophagy in tumour cells, alteration of the tumour microenvironment, increase of cancer stem cells, mutation of drug targets, reprogramming of cellular metabolism.^68^

### 3.1 Drug transport

Drug transport plays a crucial role in modulating the drug concentration in and out of cancer cells. ATP‐binding cassette (ABC) transporters are a class of membrane‐spanning proteins that play a crucial role in the transportation of anticancer drugs.^11^, ^69^ Accumulating studies have indicated that specific lncRNA play a role in regulating ABC transports in drug‐resistant tumour cells.^70^, ^71^, ^72^, ^73^, ^74^ The interaction between lncRNA and miRNA serves as a key regulatory factor in the development of chemoresistance mediated by ABC transporters. LncRNA HOTAIR is upregulated in gastric cancer cells and closely linked to the development of resistance to oxaliplatin, a commonly used chemotherapeutic agent. HOTAIR acts as a competing endogenous RNA (ceRNA) by sequestering miR‐195‐5p, resulting in the upregulation of ABCG2 expression. This molecular mechanism ultimately contributes to the development of oxaliplatin resistance.^75^ On the contrary, the presence of downregulated lncRNA has also been observed in resistant cancer cells. LncRNA GAS5 exhibits downregulation in resistant breast cancer cells, concomitant with an upregulation of ABCB1 expression level. Mechanistically, GAS5 acts as an endogenous sponge, absorbing miR‐221‐3p and subsequently inhibiting the activation of Wnt signalling pathway.^76^

### 3.2 DNA damage repair

In normal cells, DNA damage repair maintains the genomic integrity. In tumour cells, however, the continuous activation of DNA damage repair signalling plays a vital role in the failure of chemotherapy in cancer.^77^, ^78^, ^79^, ^80^ The microprotein DDUP, which is encoded by lncRNA CTBP1‐DT, exhibited a significant increase in cisplatin‐resistant ovarian cancer cells. Furthermore, there was an inverse correlation between the expression of DDUP and the response to cisplatin‐based therapy.^81^ Similarly, lncRNA SCAT7 is upregulated in response to DNA‐damaging drugs such as cisplatin. In addition, it has been established that lncRNA MALAT1 functions as a competing endogenous RNA, absorbing miR‐146a and miR‐216b. This interaction leads to the upregulation of BRCA1 expression and the preservation of the homologous recombination (HR) DNA damage repair pathway in non‐small cell lung cancer (NSCLC) cells. Overexpression of MALAT1 has been shown to protect NSCLC cells against the cytotoxic effects of cisplatin.^82^

### 3.3 Tumour microenvironment

The tumour microenvironment (TME) is an indispensable soil for tumour growth and development, which contains cancer‐associated fibroblasts, immune cells, cytokines and exosomes, among others.^12^, ^83^ A microarray analysis was conducted on the supernatant and exosomes obtained from AZD9291‐resistant lung cancer cells. The results revealed a significant increase in the expression of lncRNA MZT2A‐5 and CCDC103‐7. While only the overexpression of MZT2A‐5 could promote migration and inflammation of cancer cells, and thus contributing to AZD9291‐resistance.^84^

### 3.4 Autophagy

Autophagy is an auto‐catabolic process that permits cells to deal with stress‐coping strategies by degrading damaged organelles and accumulated proteins, which could result in cancer resistance treated with anticancer drugs.^85^, ^86^, ^87^, ^88^, ^89^ LncRNA regulates cell autophagy and contributes to chemoresistance. For example, lncRNA HOTAIR is aberrantly expressed in ovarian cancer cells, exerting regulatory control over cisplatin‐induced autophagy. Interfering HOTAIR in ovarian cancer has the potential to decrease cisplatin‐induced autophagy, offering a novel therapeutic approach for ovarian cancer treatment.^75^

### 3.5 Cancer cell stemness

Cancer stem cells (CSCs) are a population of self‐renewal cells with high tumorigenic potency. These properties can confer tumour cells with the ability to withstand harmful stressors, such as the detrimental effects of chemotherapy drugs and radiation.^90^, ^91^ LncRNA FEZF1‐AS1 and lncRNA THOR have been previously documented to modulated the stemness of cancer cells by influencing the expression of CSC markers, such as SOX9, CD44 and NANOG.^92^, ^93^ Similarly, the expression of lnc‐PKD2‐2‐3 is positively correlated with CSC markers in cholangiocarcinoma (CCA) and is markedly upregulated in stem‐like cells when compared to normal CCA cells.^94^ PKD2‐2‐3 has been found to increase the efficiency of sphere formation and enhance drug resistance to 5‐FU in CCA.

### 3.6 Glycolysis

Tumour cells prefer to consume glucose to produce lactate, even under oxygen‐rich conditions. This phenomenon is commonly known as the ‘Warburg effect’ or ‘aerobic glycolysis’.^95^ The high glycolytic flux depends on glycolysis‐associated genes, including glucose transporter type 1 (GLUT1), GLUT3, lactic dehydrogenase A (LDHA), LDHB, and hypoxia‐inducible factor 1‐alpha (HIF‐1a). This leads to the synthesis of pyruvate, alanine, and lactate.^96^, ^97^ The expression of lncRNA SNHG16 has been found to be positively correlated with glucose metabolism and resistance 5‐Fu treatment in gastric cancer.^98^ Mechanistically, SNHG16 is able to downregulate several glycolysis enzymes, including GLUT1, HK2, and LDHA, through the miR‐506‐3p/PTBP1 axis. Additionally, it has been observed that lncRNA such as SNHG7, DANCR, HAGLR and NEAT1 have the ability to modulate the expression of LDHA, a glycolysis enzyme. This modulation has been found to sensitize drug‐resistant cancer cells.^99^, ^100^, ^101^, ^102^

## 4 INTERACTION BETWEEN M6A MODIFICATION AND LNCRNA IN CANCER DRUG RESISTANCE

**FIGURE 3:**
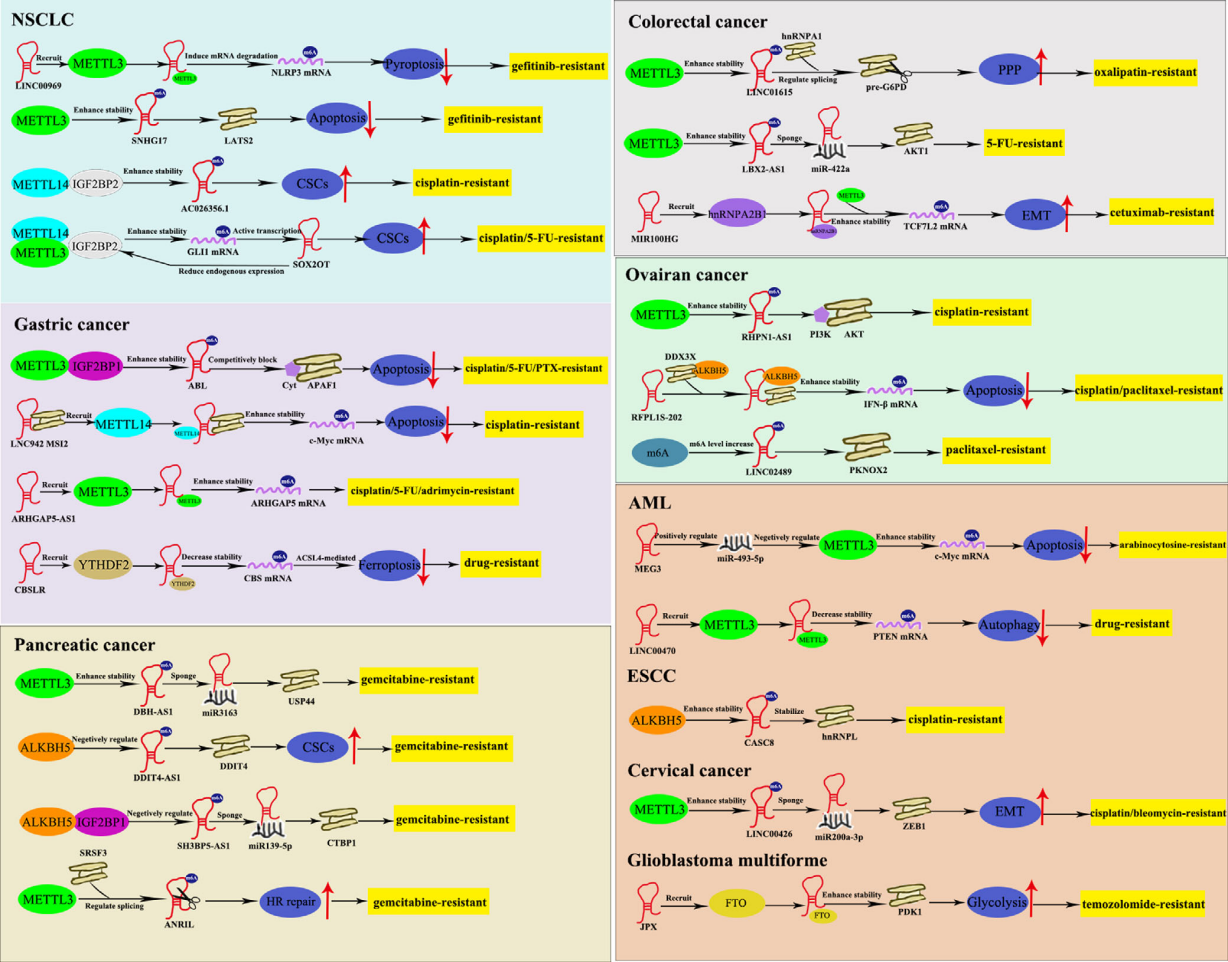
Interaction between m6A modification and lncRNA in cancer drug resistance. (1) In lung cancer: LINC00969 interacts with METTL3 and then induces NLRP3 mRNA degradation; METTL3 induces upregulation of SNHG17 by stabilizing its RNA transcript; METTL14, together with IGF2BP2, enhances AC026356.1 stability; SOX2OT facilitates METTL3/14/IGF2BP2‐mediated m6A recognition on GLI1 mRNA, thus increasing its stability. (2) In gastric cancer: ABL directly binds to m6A reader IGF2BP1, IGF2BP1 further recognizes METTL3‐mediated m6A modification on ABL; LNC942 directly binds to METTL14; ARHGAP5‐AS1 stabilizes ARHGAP5 mRNA by recruiting METTL3; CBSLR interacts with YTHDF2 to form a CBSLR/YTHDF2 complex, thus decreasing CBS mRNA stability. (3) In pancreatic cancer, METTL3 enhances DBH‐AS1 stability; ALKBH5 negatively modulates DDIT4‐AS1 expression in an m6A‐dependent manner; ALKBH5/IGF2BP1 negatively modulates SH3BP5‐AS1 stability; METTL3‐mediated m6A modification on ANRIL is essential for its splicing. (4) In colorectal cancer: METTL3 increases LINC01615 stability in a m6A‐dependent manner; METTL3 increases the stability of LBX2‐AS1; MIR100HG and hnRNPA2B1 cooperatively enhanced TCF7L2 stability. (5) In ovarian cancer: METTL3 enhances RHPN1‐AS1 stability; RFPL1S‐202 interacts with DDX3X to form RFPL1S‐202/DDX3X/ALKBH5 complex, thus increasing m6A modification on IFNB1; m6A modified LINC02489 is significantly upregulated in OC cells. (6) In myeloid leukaemia: MEG3 leads to upregulated expression of miR‐493‐5p, which targets METTL3; LINC00470 positively regulates m6A modification on PTEN mRNA via METTL3. (7) In oesophageal carcinoma: ALKBH5 increases the stability of CASC8. (8) In cervical cancer: METTL3 promotes LINC00426 stability in an m6A‐dependent manner. (9) In glioblastoma multiforme: JPX interacts with PDK1A and maintains its stability in FTO‐dependent manner. CSCs, Cancer stem cells; HR repair, DNA damage homologous recombination repair; PPP, pentose phosphate pathway; EMT, epithelial‐to‐mesenchymal transition.

Great breakthroughs have been made in the field of cancer treatment, but the strategies are not as effective as expected due to the emergence of drug resistance. The m6A modification, along with lncRNA, has been identified to play a vital role in the proliferation, migration, and invasion of various tumours, such as lung cancer, gastric cancer, cervical cancer, breast cancer, and colorectal cancer cells, among others. The MeRIP‐m6A‐seq technology enables the identification of various m6A‐modified lncRNAs and their interaction has arisen wide attention in clinical research and medical development. The potential role of m6A‐modified lncRNA as key regulators in tumour chemoresistance remains undefined. Meanwhile, m6A modification is also affected by lncRNA, suggesting that lncRNA may have an influence on the m6A recognition of downstream targets. In this section, we aim to provide novel insights into cancer treatment strategies by summarizing all the identified interactions between m6A modification and lncRNA that participate in cancer drug resistance (Table 1, Figure 3).

**TABLE 1:** Interaction between m6A modification and lncRNA in cancer drug resistance.

Cancer type	m6A regulator	lncRNA	Chemotherapeutic drug	Interaction	Mechanism	Ref
GBM	FTO	JPX	Temozolomide	JPX recruits FTO	PDK1‐mediated aerobic glycolysis	26
NSCLC	METTL3	LINC00969	Gefitinib	LINC00969 recruits METTL3	NLRP3‐mediated pyroptosis	109
	METTL3	SNHG17	Gefitinib	METTL3 enhances SNG17 stability	LATS2‐mediated apoptosis	110
	METTL3/METTL14/IGF2BP2	SOX2OT	Cisplatin/5‐FU	SOX2OT reduces endogenous expression of METTL3/METTL14	Cancer cell stemness	111
	METTL14/IGF2BP2	AC026356.1	Cisplatin	METTL14/IGF2BP2 enhances AC026356.1 stability	Cancer cell stemness	112
CRC	METTL3/hnRNPA2B1	MIR100HG	Cetuximab	MIR100HG directly recruits hnRNPA2B1	TCF7L2‐mediated EMT	67
	METTL3	LINC01615	Oxaliplatin	METTL3 enhances LINC01615 stability	G6PD‐mediated activation of PPP	118
	METTL3	LBX2‐AS1	5‐FU	METTL3 enhances LBX2‐AS1 stability	miR‐422a/AKT1 axis	119
OC	METTL3	RHPN1‐AS1	Cisplatin	METTL3 enhances RHPN1‐AS1 stability	PI3K/AKT pathway	124
	ALKBH5	RHPL1S‐202	Cisplatin/Paclitaxel	RHPL1S‐202 interacts with DDX3X/ALKBH5	IFN‐β/STAT1‐induced apoptosis	125
	‐	LINC02489	Paclitaxel	m6A modification of LINC02489 increases	PKNOX2/mTOR/PTEN axis	126
PC	ALKBH5	DDIT4‐AS1	Gemcitabine	ALKBH5 negatively regulates DDIT4‐AS1	Cancer cell stemness	128
	ALKBH5/IGF2BP1	SH3BP5‐AS1	Gemcitabine	ALKBH5/IGF2BP1 negatively regulates SH3BP5‐AS1	miR‐139‐5p/CTBP1/Wnt axis	131
	METTL3	DBH‐AS1	Gemcitabine	METTL3 enhances DBH‐AS1 stability	miR‐3163/USP44 axis	132
	METTL3	ANRIL	Gemcitabine	METTL3 is essential for SRSF3‐induced ANRIL splicing	DNA HR repair	133
GC	YTHDF2	CBSLR	‐	CBSLR recruits YTHDF2	ACSL4‐mediated ferroptosis	135
	METTL3	ARHGAP5‐AS1	Cisplatin/5‐FU/Adriamycin	ARHGAP5‐AS1 recruits METTL3	ARHGAP5‐AS1/ARHGAP5 axis	136
	METTL14	LNC942	Cisplatin	LNC942 recruits METTL14	c‐Myc‐mediated apoptosis	137
	METTL3/IGF2BP1	ABL	Cisplatin/5‐FU/PTX	METTL3/IGF2BP1 enhances ABL stability	Antagonize apoptosis by competitively block Cyt/APAF1	138
AML	METTL3	MEG3	Arabinocytosine	MEG3 positively regulates miR‐493‐5p which targets METTL3	c‐Myc‐mediated apoptosis	144
	METTL3	LINC00470	‐	LINC00470 recruits METTL3	PTEN‐mediated autophagy	145
ESCC	ALKBH5	CASC8	Cisplatin	ALKBH5 enhances CASC8 stability	hnRNPL/Bcl2/caspase3	152
CC	METTL3	LINC00426	Cisplatin/Bleomycin	METTL3 enhances LINC00426 stability	ZEB1‐mediated EMT	156

Abbreviations: AML, acute myeloid leukaemia; CC, cervical cancer; CRC, colorectal cancer; ESCC, oesophageal squamous cell carcinoma; GBM, glioblastoma multiforme; GC, gastric cancer; NSCLC, non‐small cell lung cancer; OC, ovarian cancer; PC, pancreatic cancer.

### 4.1 Lung cancer

Lung cancer is the most common malignant tumours globally and the leading cause of cancer‐related mortality in men, as well as the third leading cause of cancer death in women.^103^ The median age at the time of diagnosis is approximately 70 years.^104^ Smoking is widely recognized as the major risk factor for lung cancer and the epidemiologic patterns of this disease closely mirror the patterns of cigarette smoking. Nearly 85% of lung cancer cases are classified as non‐small cell lung cancer (NSCLC),^105^ and lung adenocarcinoma (LUAD) is the most common subtype of NSCLC, accounting for more than 40% of all cases.^105^ EGFR‐TKI, including gefitinib and erlotinib, are the first line chemotherapeutics against advanced NSCLC.^106^, ^107^ Unfortunately, acquired resistance has developed, leading to tumour recurrence and metastasis.^108^ Therefore, it is necessary to figure out the mechanism underlying EGFR‐TKIs resistance and explore new strategies to reverse this resistance. Bioinformatic analyses revealed a significant upregulation of LINC00969 expression, which was found to promote resistance to gefitinib in chemoresistant lung cancer cells. Mechanistically, the interaction between LINC00969 and EZH2 and METTL3 observed. This interaction transcriptionally regulated the level of H3K27me3 in NLRP3 promoter region and post‐transcriptionally modified the m6A level of NLRP3. Consequently, the repression of NLRP3 resulted to gefitinib resistance in NSCLC through the suppression of the classical pyroptosis signalling pathway.^109^ Similarly, the expression of lncRNA SNHG17 was increased in gefitinib‐resistant lung cancer cells and contributed to the development of gefitinib resistance in LUAD. Mechanistically, METTL3‐mediated m6A modification enhanced the stability of SNHG17, leading to an upregulation in its expression. The increased SNHG17 promoted gefitinib resistance in LUAD by epigenetically repressing LAST2.^110^ In addition, GLI1 served as a key terminal factor in SHH pathway, playing a vital role in cancer cell stemness and drug resistance. In addition to the upregulation of GLI1, lncRNA SOX2OT was also found to be positively regulated in drug‐resistant NSCLC cells. Further mechanistic investigation revealed that SOX2OT played a role in facilitating METTL3/14/IGF2BP2‐mediated m6A recognition on GLI1 mRNA, thereby contributing to chemoresistance in lung cancer.^111^ Moreover, the stability of AC026356.1 was enhanced and its expression level was increased by the combined action of METTL14 and IGF2BP2. The upregulated AC026356.1 has been shown to play a crucial role in the maintenance of cancer cell stemness by activating the Wnt signalling pathway^112^ in LUAD.

The above evidence provides a comprehensive illustration of the significant role played by m6A‐modified lncRNA, as well as lncRNA‐regulated m6A regulators in the development of TKI‐resistance in NSCLC. This resistance is primarily attributed to the repression of apoptosis and the augmentation of stemness.

### 4.2 Colorectal cancer

Colorectal cancer (CRC) accounts for approximately 10% of annually diagnosed cancers and cancer‐related deaths worldwide. It is the second most common cancer in women and the third most in men. Patients under the age of 50 have been observed to experience a concerning increase.^113^ Drug resistance, which is observed in patients with CRC, has emerged as a significant factor leading to poor treatment outcomes.^114^ Epithelial‐to‐mesenchymal transition (EMT) is a dynamic biological process.^115^ Epithelial cancer cells undergoing EMT are characterized by enhanced drug resistance. MIR100HG is a miRNA‐host transcript, generating about 17.5% of miRNA.^116^, ^117^ MIR100HG and hnRNPA2B1, a m6A reader protein, cooperatively increased TCF7L2 mRNA stability in a m6A‐dependent manner, thus inducing cetuximab resistance in CRC patients.^67^ Cancer cells have developed various manners to cope with nutrient starvation. LINC01615 was found to play a protective role in CRC cells under serum‐deprived conditions by promoting cell survival rate through the activation of the pentose phosphate pathway (PPP). Mechanistically, the process of serum starvation led to the degradation of METTL3, consequently leading to an elevation in the stability and expression level of LINC01615. LINC01615 was found to enhance the expression of G6PD, an essential enzyme in the PPP, through its competitive binding with hnRNPA1 and its facilitation of pro‐G6PD mRNA splicing.^118^ In addition, the expression of lncRNA LBX2‐AS1 level was upregulated in CRC tissues, and this upregulation was observed to be positively associated with 5‐FU resistance. Mechanistic investigations suggested that METTL3‐mediated m6A modification increased the stability and expression level of LBX2‐AS1.^119^

Thus, interaction between m6A modification and lncRNA results to chemotherapeutics resistance in CRC tissues via promoting EMT/DNA damaging repair.

### 4.3 Ovarian cancer

Globally, ovarian cancer (OC) is the seventh most common cancer in women and the eighth leading cause of cancer‐related mortality. Ovarian cancer is a relatively uncommon occurrence in women under the age of 40, but its incidence rises steadily after the age of 40 and reaches the peak in the late 70s. Women who have a family history are at higher risk of the disease.^120^ The administration of cisplatin in OC patients lasts for many years.^121^, ^122^ However, the development of cisplatin resistance poses a big obstacle for OC therapy owing to a 5‐year survival rate <30%.^123^ Researchers transfected lncRNA RHPN1‐AS1 overexpression plasmids into cisplatin‐resistant OC cells and found that the upregulation of RHPN1‐AS1 significantly promoted the progression, migration and invasion of OC cells in vivo. Further investigation has demonstrated that METTL3 promoted RHPN1‐AS1 stability via m6A modification, thus resulting to the development of cisplatin resistance in OC cells via PI3K/AKT pathway.^124^ Liu's study revealed that lncRNA RFPL1S‐202 played a significant role in enhancing the apoptotic effects of paclitaxel and cisplatin on OC cells. Mechanistically, RFPL1S‐202 physically interacted with DDX3X/ALKBH5, thereby decreasing the expression of IFN by increasing the m6A modification of IFNB1.^125^ In addition, when comparing to primary OC tissues, LINC02489 expression decreased dramatically in metastatic and chemoresistant OC tissues. Overexpression of LINC02489 would inhibit proliferation, invasion, and migration of drug‐resistant OC cells through the upregulation of PKNOX2 m6A modification.^126^

To conclude, the m6A modification plays a significant role in enhancing the stability of relevant lncRNA, thereby leading to the development of cisplatin/paclitaxel resistance in OC tissues through various different signalling pathway.

### 4.4 Pancreatic cancer

Pancreatic cancer (PC) ranks as the seventh leading cause of cancer‐related death worldwide, exhibiting a mere 5‐year survival rate of only 9%.^127^, ^128^ The incidence of PC is typically rare before the age of 40. However, due to the shifting age structure of the global population, there has been an increase in the incidence of PC.^129^ To date, the aetiology of PC is still insufficiently known, although certain risk factors have been identified, such as smoking, obesity, genetics, and diabetes. Gemcitabine (GEM) improves the life quality and increases the survival rates in patients with advanced pancreatic cancer.^130^ However, resistance to gemcitabine significantly limits its application in clinic treatment. ALKBH5 exerted a negative regulatory effect on the expression of lncRNA DDIT4‐AS1, thus suppressing the sensitivity of PDAC cells to GEM via mTOR pathway.^128^ Similarly, Lin illustrated that lncRNA SH3BP5‐AS1 was significantly upregulated in gemcitabine‐resistant pancreatic cancer cells and this upregulation was found to be associated with a poor prognosis for PC patients. Mechanistically, the stability of SH3BP5‐AS1 was negatively regulated by ALKBH5/IGF2BP1‐mediated m6A modification.^131^ Furthermore, increased m6A‐modified lncRNA DBH‐AS1 suppressed GEM resistance in pancreatic cancer. DBH‐AS1 increased sensitivity of PC cells to GEM by sponging miR‐3163 and upregulating USP44 in PC cells.^132^ Additionally, it is considered the occurrence of abnormal splicing may lead to GEM chemoresistance in PC cells. For instance, the splicing of lncRNA ANRIL was found to be regulated by SRSF3, and that m6A modification on ANRIL was essential for its splicing process. ANRIL‐L would then regulate pancreatic cancer cell chemoresistance by enhancing DNA homologous recombination (HR) repair.^133^

The mentioned evidences reveal that m6A‐modified lncRNA has the potential to induce GEM resistance in PC tissues via promoting cancer cell stemness or enhancing DNA damage repair.

### 4.5 Gastric cancer

Gastric cancer (GC) is the fifth most common cancer and the third leading cause of cancer‐related death worldwide. GC occurs more frequently in males and there has been a recent increase in younger patients from high‐income countries.^134^ Chemotherapy is the first‐line adjuvant therapy for GC patients, but chemoresistance poses a major obstacle for GC clinic therapy.^134^ Therefore, exploring mechanisms underlying chemoresistance in GC might improve the clinical outcome and increase the survival rates of patients with GC. Researchers have identified that lncRNA‐CBSLR played a protective role in GC cells by preventing ferroptosis, which ultimately results in chemoresistance in GC. Mechanically, the interaction between CBSLR, YTHDF2, and CBS contributed to the downregulation of CBS mRNA stability and expression level. Decreased CBS levels further reduced the methylation of the ACSL4 and leaded to the degradation of ACSL4, thus contributing to ferroptosis‐induced resistance in GC.^135^ Additionally, the expression of lncRNA ARHGAP5‐AS1 was increased in chemoresistant GC cells, and knockdown of ARHGAP5‐AS1 could reverse the chemoresistance in these cells. Mechanistically, ARHGAP5‐AS1 activated the transcription of ARHGAP5 in the nucleus and stabilized ARHGAP5 mRNA in the cytoplasm by recruiting METTL3.^136^ Moreover, LNC942 was found upregulated in chemoresistant GC cells. Functional studies indicated that LNC942 stabilized c‐Myc mRNA stability, leading to the chemoresistance in GC cells by inhibiting apoptosis and promoting stemness.^137^ LncRNA ABL was upregulated in GC cells as well. ABL directly bound to IGF2BP1 through its KH1/2 domain, and IGF2BP1 subsequently recognized METTL3‐mediated m6A modification on ABL.^138^

In summary, it has been observed that specific lncRNA directly recruits m6A regulators and strengthens m6A recognition of downstream targets, thus mediates cisplatin‐resistance in GC tissues mainly via blocking apoptosis.

### 4.6 Other cancer

Acute myeloid leukaemia (AML) is a relatively aggressive myeloid malignancy with strikingly heterogenous outcomes.^139^, ^140^, ^141^ AML remains a relatively uncommon form of cancer, however, it accounts for one third of all leukaemias diagnosed. Arabinocytosine (AraC) is one of the most effective chemotherapeutics for AML treatment, however resistance to AraC poses a persistent obstacle for increasing clinic treatment effect.^142^, ^143^ Expression of lncRNA MEG3 and miR‐493‐5p were found to be relatively decreased in chemoresistant AML cells. MEG3 positively regulated miR‐493‐5p expression which directly targeted METTL3.^144^ Moreover, it was found that LINC00470 positively regulated m6A modification of PTEN mRNA via METTL3 and inhibited PTEN expression, thus leading to the suppression of autophagy and the promotion of chemoresistance in AML cells.^145^

Glioblastoma multiforme (GBM) is the most common form of brain cancer and one of the most aggressive cancers found in humans, with a survival rate of 5.5% at five years.^146^ Temozolomide (TMZ) improves the life quality of GBM patients. However, the effectiveness of TMZ is significantly hindered by the development of inherent or acquired TMZ resistance, which seriously limits its clinic application.^147^ LncRNA JPX expression was significantly upregulated in GBM tissues. Further mechanism studying revealed that JPX participated in the proliferation of GBM cells and their resistance to TMZ. Mechanistically, JPX interacted with PDK1A mRNA and maintained its stability in an FTO‐dependent manner.^26^

Oesophageal carcinoma (EC) is the sixth leading cause of cancer‐related mortality and the eighth most common cancer worldwide, with the overall 5‐year survival ranges from 15% to 25%. Oesophageal squamous cell carcinoma (ESCC) is the most common subtype of EC.^55^, ^148^, ^149^ Chemotherapy is the third major treatment for ESCC,^150^ but acquired drug resistance limits the clinic effect.^151^ LncRNA CASC8 was significantly upregulated in ESCC tissues, accompanied with upregulated cell proliferation and cisplatin sensitivity. Mechanistically, the upregulation of CASC8 in ESCC was associated with ALKBH5‐mediated m6A demethylation via regulating its stability.^152^

Cervical cancer (CC) is the fourth leading cause of cancer‐related death in women, with poor clinic outcomes.^153^ The epidemiological patterns of CC are often coined a ‘disease of disparity’, because of the significant disparity in incidence and mortality between low‐income and middle‐income countries.^154^ Combination of cisplatin and paclitaxel is a standard chemotherapeutic strategy to treat recurrent or metastatic cervical cancer, however, chemoresistance has a negative influence on the clinical effect.^155^ Bioinformatics analysis showed LINC00426 was upregulated in CC. Overexpression of LINC00426 in CC cells induced resistance to cisplatin and bleomycin. Mechanistically, METTL3 promoted the expression of LINC00426 by m6A modification, thus affecting proliferation, migration, and invasion of CC cells by regulating the expression of some EMT markers.^156^

To conclude, the mutual regulation between m6A modification and lncRNA cooperatively contribute to the drug resistance in AML, ESCC, CC and GBM. Blocking the interplay between m6A modification and lncRNA may represent novel therapeutic targets for cancer treatment strategies.

## 5 CONCLUSION AND FUTURE PERSPECTIVE

Resistance to therapy continues to be the biggest challenge in cancer today. There exist multiple underlying mechanisms of resistance that can eventually lead to death. Solving the issue of resistance appears to be an unachievable objective. In this review, we aim to provide a summary of the existing literature on the reciprocal regulation between m6A modification and lncRNA, as well as their respective roles in cancer drug resistance. On one hand, the m6A modification has a significant impact on the structure, transcription, biogenesis, stability, and regulatory networks of lncRNA. On the contrary, m6A regulators are also affected by specific lncRNA, thereby playing a role in the installation, removal and identification of m6A modifications on RNA molecules. Owing to the development of MeRIP‐m6A‐seq technology in 2012,^40^ a significant number of m6A‐modified lncRNA has been identified in the field of chemoresistance. This discovery has opened up new avenues for targeting these modified lncRNAs in order to enhance the sensitivity of chemotherapeutic agents.

However, with a series of miRNA‐based therapeutics applied in phase II or III clinical treatment, the progress in lncRNA‐related research has primarily centred on clarifying the mechanisms of chemoresistance, without any practical application in clinical settings so far. Targeting lncRNA and its associated RBPs provides new directions for clinical intervention. Additionally, recent advancements have suggested that certain lncRNA may have the ability to encode microproteins.^33^ However, further investigation is required to determine whether m6A modification involves in the production of these microproteins. The investigation of the role of these micropeptides in the development of drug resistance in cancer warrants further examination in future studies. Additionally, it is worth noting that so‐far, there is a lack of evidence that specifically examines the role of m6A‐related lncRNA in regulation of the tumour microenvironment. Perhaps, the limitation of MeRIP‐m6A‐seq technology hinders the comprehensive characterization of the tumour microenvironment across various cell types.

So far, studies on interaction between m6A modification and lncRNA in the context of chemoresistance are still low in number. More extensive research is required to enhance the clinical outcomes of patients on a global scale.

## AUTHOR CONTRIBUTIONS

Jin Yizhou: design and conception, search of literature, manuscript writing, creation of figure and table, and final approval of the manuscript. Fan Zhipeng: design and conception, manuscript revising, financial support, and final approval of the manuscript. All authors read and approved the final paper.

## FUNDING INFORMATION

This work was supported by grants from the National Natural Science Foundation of China (82130028 to Z.P.F.), National Key Research and Development Program (2022YFA1104401), CAMS Innovation Fund for Medical Sciences (2019‐I2M‐5‐031 to Z.P.F.) and grants from Innovation Research Team Project of Beijing Stomatological Hospital, Capital Medical University (No. CXTD202204 to Z.P.F.).

## CONFLICT OF INTEREST STATEMENT

The authors declare no potential conflicts of interest.

## PERMISSION TO REPRODUCE MATERIAL FROM OTHER SOURCES

Reproduced under terms of the CC‐BY licence.

## Data Availability

All data used to support the findings of this study are included within the article.
